# Causal impact evaluation of occupational safety policies on firms’ default using machine learning uplift modelling

**DOI:** 10.1038/s41598-024-60348-4

**Published:** 2024-05-06

**Authors:** Berardino Barile, Marco Forti, Alessia Marrocco, Angelo Castaldo

**Affiliations:** 1 Centre for Intelligent Machines, Department of Electrical and Computer Engineering, McGill University, Montréal, Canada; 2 MILA (Quebec AI Institute), Montréal, Canada; 3 Department of Juridical and Economic Studies, Sapienza University of Rome, Rome, Italy

**Keywords:** Computer science, Applied mathematics, Scientific data, Statistics

## Abstract

It is often undermined that occupational safety policies do not only displace a direct effect on work well-being, but also an indirect effect on firms’ economic performances. In such context, econometric models dominated the scenes of causality until recently while Machine Learning models were seen with skepticism. With the rise of complex datasets, an ever-increasing need for automated algorithms capable to handle complex non-linear relationships between variables has brought to uncover the power of Machine Learning for causality. In this paper, we carry out an evaluation of a public aid-scheme implemented in Italy and oriented to support investment of small and medium enterprises (SMEs) in occupational safety and health (OSH) for assessing the impact on the survival of corporations. A comparison of thirteen models is performed and the Individual Treatment Effect (ITE) estimated and validated based on the AUUC and Qini score for which best values of 0.064 and 0.407, respectively, are obtained based on the Light Gradient Boosting Machine (LightGBM). An additional in-depth statistical analysis also revealed that the best beneficiaries of the policy intervention are those firms that experience performance issues in the period just before the interventions and for which the increased liquidity brought by the policy may have prevented default.

## Introduction

Public policies on occupational safety and health (OSH) aim primarily at improving working conditions. Usually, this objective is set at constitutional level and through other regulatory sources, both at the European and national level. The European Agency for Safety and Health at Work underlines the need for a mixed approach in addressing the challenge of improving health and safety conditions in the workplace by relying on both legal regulation and its enforcement (sticks), as well as economic incentives (carrots). Nevertheless, in Europe the use of *carrots* is much less widespread than *sticks*, and the former, even where implemented, is not provided as a structural policy tool.

In Italy, since 2010, the Italian National Institute for Insurance against Accidents at Work (INAIL) has launched a State-aid scheme (ISI calls) to support firms’ (especially SMEs) investments to improve Occupational Safety and Health (OSH) performance. Under a theoretical perspective, OSH policies do not only displace a direct effect on work well-being, but also an indirect effect on firms’ economic performance^1–6^. Following Uegaki et al.^7^, we can identify four labels to denote four proxies of the measure of productivity that links health to firm performance and, hence, in our perspective, to survival: (1) sick leave; (2) compensated sick leave; (3) limited or modified operational activities; and (4) working-presenteeism^.^ At the operational level, this means that when workplace accidents occur there is a decrease in production (imputable to days loss, and equipment damages) and/or a deterioration in product quality; moreover, in the case in which workers are still at work even though not fully healed, they could operate with a lower productivity. In both cases, the result is a loss of part of the profits and productivity that would have been potentially obtained, considering the optimal scenario of production at full capacity and without defects^6^.

Understanding the economic perspective is particularly important in the context of OSH: on the policy-makers side, unsafe or unhealthy working conditions lead to negative externalities with respect to the costs that workers and firms bear. Indeed, injuries and professional illness related to working population are accompanied by significant socio-economic burdens^7^, usually in the form of costs (monetary and non-monetary) for third parties—i.e., families, relatives, and society^8–10^. On the firm side, investments in OSH are usually made (besides ensuring the minimum legal standards) at the discretion of managers and shareholders, for whom economic gains are a crucial factor in the economic attractiveness of OSH^11^. However, firms, especially small and medium enterprises (SMEs) do not hold a complete set of information on the impact of occupational injuries and illnesses on business performance^12–15^; the concern is that the lack of awareness may lead to underinvestment compared to the “ideal” level^10,11,16–18^. To realize how health and safety performance adapt strategically to the firm's operational performance, investments in safety should be communicated as an added value rather than a cost^4^.

In the context of public policy evaluation, a significant and innovative contribution has been provided in the field of Causal Machine Learning (CML). Within this wide and steadily growing methodological set up, uplift modelling, also known as true-lift modelling, represents a technique that directly models the incremental impact of a treatment at an individual level. Such a technique represents a powerful tool to investigate public policy interventions due to their capability to partition the overall causal impact onto the single statistical units. In fact, in most applications it is also interesting to look beyond the average effects to understand how the causal impact vary with observable characteristics^19^. More precisely, instead of focusing on an output variable, uplift modelling focusses on the change in the output variable caused by an intervention. In recent years, the use of CML based on uplift modelling has sparked the interests of many researchers in different fields of studies such as education, social welfare, and public health among others. The widespread of CML has led to numerous scientific publications, especially in the biomedical sector where most of the scientific contributions are presented^20^. Interestingly, the use of uplift modelling has started to become widely used also in private companies due to its capability to identify the most responsive customers with respect to a specific outcome (i.e., increase of sales). In the context of public policy interventions, uplift modelling is a relatively new topic due to the difficultly in acquiring datasets.

In this paper we employ classical CML methods for the identification of the Individual Treatment Effect (ITE) originated from the ISI Calls implemented in Italy by INAIL. To the best of our knowledge, this work represents the first attempt to assess the impact of OSH direct aid-scheme on firms default by means of CML, and one of the first evaluation analysis of the effect exerted by any other public incentive scheme on firms’ failure. The advantage of using ML approaches for causal inference is well documented in the literature^21^. Contrary to classical econometric approaches where the Average Treatment Effect (ATE) represents the main objective of the investigation, CML may open new perspectives under the idea that not all participants benefit from the policy intervention to the same extent^19^. This new type of public policy evaluation provides valuable information to the policymaker by providing not only the traditional ATE of the policy, which measure the effectiveness of the public intervention, but also low-level information of the subgroups of participants that most benefitted from the intervention. In light of these objectives, this paper addresses the problem of estimating the ITE onto the second-round effect produced by the ISI initiative launched by INAIL toward firms operating in Italy by employing ML techniques based on survival data.

The paper is structured as follows. In Section "Causal machine learning for public policy evaluation" we present the main motivations and the added value for public policy evaluation provided by the CML approach. Section "Evaluation design strategy overview" provides an overview of the policy evaluation strategy that we implement. In particular, details regarding the participation and legal procedures implied by the public tender are introduced. Specifically, the first analysis aims to evaluate the policy by recurring to the standard approach of classification between censured and failed corporations. The second analysis aims at extending the classification task by considering time-dependent data using survival models specifically designed for handling censored observations. Finally, the uplift model approach is described with the aim to evaluate the causal impact of the policy through the estimation of the ITE for each firm. Moreover, in Section "Statistical and machine learning models", the statistical and machine learning models are discussed. In Section "Experimental set-up", our main experimental set up is presented. Furthermore, in Section "Results and discussion", we present our main findings and the relative discussion. Finally, Section "Conclusion and main policy implications", draws the main conclusions and policy implications.

## Causal machine learning for public policy evaluation

Machine Learning (ML) methods have received lot of attention in recent years and these types of algorithms are primarily geared to make predictions. Conversely, empirical researchers conducting policy evaluations are primarily interested in the estimation of the causal effect by trying to answer counterfactual questions (what would have happened in the absence of a policy?). However, such counterfactual questions are difficult to be answered due to the “*fundamental problem of causal inference*”^22^, which impedes the classical usage of supervised approaches. Nonetheless, in the last decade, major innovations have taken place incorporating supervised ML tools into estimators for causal parameters such as the ATE^23^. Although the adoption of these methods in economics have been viewed with skepticism, they are now beginning to be widely used in empirical work and are driving a rapidly increasing interest under a methodological point of view^24^. The methods developed in the ML literature have been particularly successful in big data settings. Chernozhukov et al.^25^, considered one of the most important contributions in the CML literature, proposes an orthogonal score for the target low-dimensional parameter, such as regression coefficients, average treatment effects and average lifts, by combining auxiliary and main ML predictions. Their method, called "Double ML", is based on the idea of estimating primary and auxiliary predictive models as solution to the regularization bias introduced by naively plugging ML estimators into equations. Such an approach has been widely recognized as a useful framework for conducting a flexible and comprehensive program evaluation exercise. Based on this work, several other evaluation strategies have been implemented. Knaus^26^ proposes the use of a CML-based method to provide a comprehensive and computationally appropriate evaluation of four programmes of the Swiss Active Labor Market Policy. This work illustrates the potential of CML-based methods for program evaluations under unconfoundedness and provides a potential blueprint for similar analyses. Additionally, Fan *et al.*^27^ proposed new nonparametric estimators for the reduced dimensional Conditional Average Treatment Effect (CATE), given the unconfoundedness assumption. In the first stage, the nuisance functions necessary for identifying CATE are estimated by ML models, allowing the number of covariates to be equal or larger than the sample size. The second stage consists in a low-dimensional local linear regression, reducing CATE to a function of the covariates of interest. Moreover, several works have successfully used CML methods for public policy evaluation. For example, Davis *et al.*^28^ propose the use of a CML model (i.e., causal random forest) for the evaluation of two randomized experiments aiming to offer a supported summer job to different categories of Chicago youth. With the use of CML model they show that the program consistently reduces violent crime arrests and uncover heterogeneity in employment impacts that standard methods would miss. In particular, they disentangle these effects by selecting the characteristics of the most responsive beneficiaries by pinning down the heterogeneous impact of the policy. Ballestar *et al.*^29^ propose the use of multilevel ML model to investigate the effects produced by economic monetary incentives program for promoting research and development in the Region of Madrid (Spain) between 2005 and 2010. In particular, the authors implement a multilevel ML approach by applying a stratification in the first stage and a non-supervised ML method in the second stage.

Although the use of CML has been exponentially increasing in the last decades, to the best of our knowledge no previous studies have implemented uplift modeling techniques to evaluate public financial subsidies for enhancing firms’ survival. For this reason, in this work we proposed a thorough evaluation of the state-aid monetary incentive scheme (ISI) initiative launched by the Italian National Institute for Insurance against Accidents at Work (INAIL) on the secondary round effect of the policy aiming at improving the likelihood of firm survival in Italy thanks to direct financial grants.

## Evaluation design strategy overview

In this section, a thorough explanation of the dataset used in this work is provided. In Section "Dataset and policy description", details regarding the participation and legal procedures implied by the public tender are introduced. In Section "The basement of our analysis: firms failure prediction" the first and second objectives of this study are discussed. Specifically, the first analysis aims to perform the standard approach of classification between censured and failed corporations. The second analysis provides an extension to the classification task by considering time-dependent data using survival models specifically designed for handling censored observations. Finally, in Section "Uplift modelling: a theoretical explanation" the uplift model approach is described with the aim to evaluate the causal impact of the policy through the estimation of the ITE for each firm.

### Dataset and policy description

**Figure 1:**
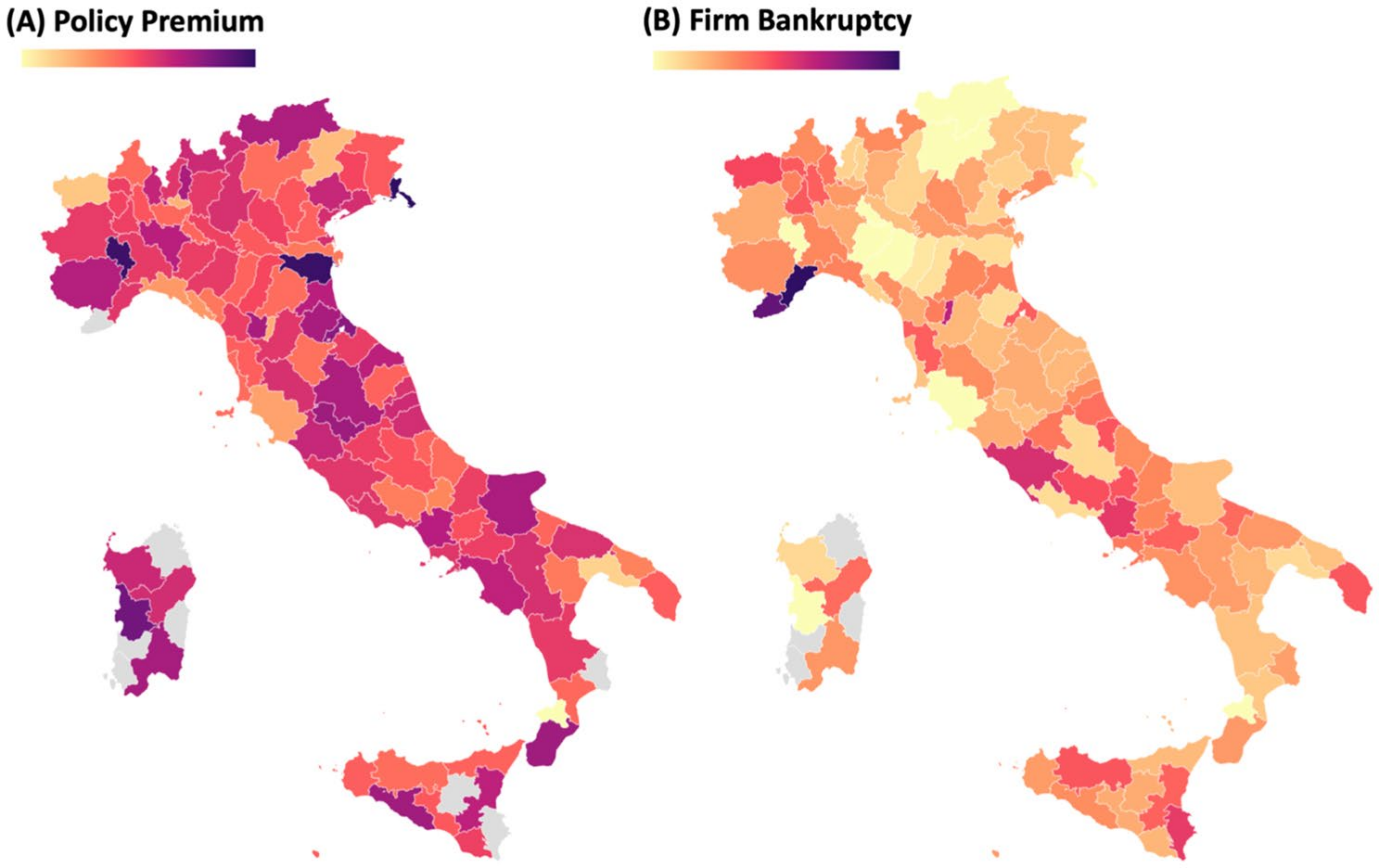
Geographical distribution of the policy premium (grant) provided by ISI 2013 and of the probability firms’ of bankruptcy.

The Italian National Institute for Insurance against Accidents at Work (INAIL) aims to reduce the phenomenon of occupational accidents and illnesses. INAIL’s activity can be divided into three branches: (1) prevention of occupational risks, (2) information, (3) training and assistance in occupational safety and health. Concerning the first branch of prevention activities, INAIL implements and promotes the protection of workers also through initiatives that provide economic support to companies; the aims of these activities are to contribute to the reduction of accidents and to develop a proper safety culture in the country. To this end, on the ground of Legislative Decree n. 81/2008, since 2010, INAIL launched the ISI initiative, which aims to provide direct subsidies to companies for supporting the implementation of projects oriented to improve the levels of health and safety at work. The economic incentives have the form of a non-repayable grant. In Europe there is no similar policy either in terms of resources allocated (over EUR 2 billion from 2010 to 2019) or in terms of structural and systemic character taken, which represents a unique characteristic of this work. In Fig. 1, the distribution of the average policy premium (A) received by each firm and their probability of default (B) is depicted stratifying observations by national provinces. The complete dataset used in this work is obtained by collecting data from three different information flows: the streams related to participants in the ISI call and to work insurance data, both provided by INAIL, plus the AIDA flow for financial information of the monitored companies. The merge between these databases is carried out considering a unique Identification Number (ID) which allowed to perform an exact matching between the three sources of data. It should be noted that the regulation precludes participation to all companies that won the call in previous editions.

In Table 1, the statistics for the Treated and control group of firms participating to the call is reported. Cleaning operations of the dataset only involved the exclusion of companies that participated and won the ISI calls in editions after 2013 as well as the dropout of the company with missing financial data. These operations are necessary to isolate the effects of the policy and guarantee feasibility of the analysis. For the purpose of the present study, the Treated and control groups are identified by exploiting the characteristics of the policy design. In particular, the ISI call is promoted on the base of a fixed amount of financial resources that are allocated to participants following the “*first-come, first-served*” principle, also called click-day. This represents a common scenario for evaluating public policy interventions due to the resemblance to a Randomized Control Trial (RCT) study. In fact, a firm which was successful and one which was not were separated by such a short period of time that the selection can be safely considered as random. Consequently, the intention to treat combined with the short amount of time (hundredths of a second) that discriminate among being treated or not, ensures that no observed or unobserved confounding features can be related to the treatment assignment. Firms were sorted based on the time in which the application was received, which represents the only determinant for treatment. The Treated group was identified considering the maximum number of firms that could be financed based on the pre-determined amount of resources available. By following this scheme, it can be reliably assumed that two firms applying to the tender have the same chance of getting Treated, thus approaching an RCT scheme. Nonetheless, we formally tested this assumption and formal evidence supports our identifying strategy.

**Table 1:** Treatment and Failure statistics of firms participating to the tender.

Status	Fail	Censored	Total
Treated	60	1240	1300
Non-treated	497	5541	6038
Total	557	6781	7338

To better understand the dataset in hand, Table 2 shows the distribution of Treated and Non-Treated firms partitioned based on the Ateco 2007 classification of economic activities. It is important to notice that since the main goal of the policy is to prevent accidents at work and promote safe cultural habits, special attention should be paid to sectors such as Manufacturing, Construction as well as Transportation and storage since these sectors include jobs more prone to injuries. Not surprisingly, this is reflected in the distribution of Treated and Non-Treated firms with 73.6% of companies classified in these three sectors.

**Table 2:** Distribution of Treated, Non-Treated and Bankruptcy Firms based on the Ateco 2007 classification of economic activities.

Ateco	Firms	Treated	Non-treated	Failed	% Failed
Agriculture, forestry, and fishing	107	6	101	8	7.48%
Mining and quarrying	102	31	71	6	5.88%
Manufacturing	3276	608	2668	215	6.56%
Electricity, gas, steam, and air-conditioning supplying	15	2	13	0	0.00%
Water supply, sewerage, waste management and remediation	145	27	118	11	7.59%
Construction	1890	362	1528	169	8.94%
Wholesale and retail trade	863	143	720	70	8.11%
Transportation and storage	239	42	197	22	9.21%
Accommodation and food service activities	106	9	97	13	12.26%
ICT	45	9	36	5	11.11%
Financial and insurance activities	2	0	2	0	0.00%
Real estate activities	61	10	51	6	9.84%
Professional	86	8	78	6	6.98%
Administrative and support service activities	144	18	126	11	7.64%
Education	10	0	10	2	20.00%
Health services	44	6	38	3	6.82%
Arts, entertainment, and recreation	25	3	22	2	8.00%
Other services	35	9	26	1	2.86%
Activities of households	1	0	1	0	0.00%
Other unclassified activities	142	7	135	7	4.93%
**Total**	**7338**	**1300**	**6038**	**557**	**7.59%**

Finally, in order to compare the financial status of beneficiary (Treated) and non-beneficiary (Non-Treated) firms, Table 3 highlights the comparison of the most important balance sheet variables describing the financial condition of firms participating to the tender. By comparing Treated and Non-Treated in the period before the policy implementation, the two groups resulted comparable, in-distribution, providing a further confirmation that the policy design allowed for an effective randomization and little to no bias between groups.

**Table 3:** Descriptive statistics of treated and non-treated firm’s financial information comparing before (2011–2014) and after (2016–2019) policy intervention.

Feature	Before		After	
	Treated	Non-treated	Treated	Non-treated
Monetary values				
Assets	4262.85	3842.44	4973.73	4514.24
Production	3813.29	3411.20	4582.01	4188.10
EBITDA	292.93	259.73	412.86	336.87
Working Capital	2915.15	2374.32	3354.24	2911.53
Net Assets	1215.01	1161.43	1618.04	1466.62
Production Costs	3650.49	3479.65	4334.74	4000.53
Net Income	67.40	46.92	149.80	100.86
Wage	438.09	412.12	549.22	509.23
Total Debts	2778.47	2437.92	3004.98	2752.18
Revenues	219,415.13	204,017.90	231,725.37	215,085.88
Financial Index				
ROE	9.35	8.60	10.61	8.64
ROS	4.83	4.23	4.98	4.44
ROA	5.15	4.53	4.82	3.55
Debt over Equity	0.74	0.65	0.03	0.06
Liquidity	0.98	0.97	0.09	0.07
Cost of Money	5.89	5.80	-0.63	-0.31
Short-term Debt	0.90	0.89	-0.05	-0.03
Long-term Debt	0.10	0.11	0.05	0.03

Monetary values are expressed in thousands of Euros.

### The basement of our analysis: firms failure prediction

Of particular interest in our study is that Italy represents a compelling case where firms are negatively affected by poor enforcement of existing regulations and excessive judicial delays^30^. These features could weaken firms' incentive to undertake investments^31^ both in general and in OSH specifically. In this sense, carrot policies (i.e., direct and indirect public aid schemes) to support OSH investment—by providing direct financial subsidies to SMEs for tangible and intangible OSH investments—could reduce the risk of underinvestment in OSH and allow for an improvement in firms' economic performance. In this context, we focus our attention on the evaluation of a unique public policy intervention to assess the impact of OSH direct aid-scheme on firms’ default.

In order to predict corporate failures, both discriminative and survival ML models are considered. Specifically, for the task of firms’ classification, a standard binary discriminative task is performed in order to automatically partition firms between failed and censored observations. Both statistical and ML models are used for the analysis.

Firstly, Lasso and Ridge regression models are used to investigate if standard statistical approaches, based on L1 and L2 regularization techniques respectively, may handle the task of bankruptcy classification even in cases were a large number of regressors and collinearity between columns is present.

In particular, these models are used as benchmark to which we compared state of the art ML techniques based on boosting trees algorithm such as LightGBM, XGBoost and CatBoost (see section "statistical and machine learning models").

Furthermore, to compare our results with the existing literature, SVM and RF models are considered and explained in Section "Machine learning (survival): random forest (survival), support vector machine (survival) and gradient boosting survival". Nonetheless, such a naive approach of predicting financial failure does not consider the time at which the failures occurred. For this reason, to overcome this limitation, we also rely on three statistical-based models (CPHM, WeibullAFT, AAH) and three ML-based algorithms (RFSurvival, SVMSurvival, GBSurvival), specifically designed for survival analysis.

It is worth noting that for the task of predicting corporate failure, both binary classification and survival based prediction tasks represent valid approaches. The interest in adding a comparison with traditional “non-survival-based” algorithms hinges on the opportunity to provide a probabilistic interpretation of the results^32^. This is particularly appealing in the context of public policy evaluation where the policy maker is interested in understanding the overall efficacy of the treatment (i.e. was the policy successful?). Additionally, due to the randomization obtained by policy design, group level effects, such as ATE, can be easily obtained with traditional econometric methods and compared with ML-based results. Conversely, for the task of estimating ITE, comparing binary with survival model might provide a more compelling interpretation of the results.

Balance sheet data as well as custom data from the policy intervention are used considering a time interval between 2011 and 2019. The time component was considered in terms of additional regressors, thus producing a dataset in a “*wide*” format. It is important to highlight that only the years between 2011 and 2016 are used for training to avoid leakage during the evaluation phase. No financial failures are observed within this time frame. For the binary classification case, we labeled with “1” all the firms experiencing financial failure between the years 2017 and 2019 (disbursements window), “0” otherwise. Conversely, for survival-based models, the task is not reduced to a binary problem and both time and censored information are exploited. Specifically, the survival time is calculated as time difference (in days) between the official starting date of the policy (29 May 2014) and the year in which the failure occurred. For censored observations, the censoring time is considered instead.

### Uplift modelling: a theoretical explanation

Causal uplift modeling concerns the estimation of the net effect of a treatment on an outcome of interest at the instance level (ITE)^33^. This causal inference task is encountered in the literature under various names such as heterogeneous treatment effect estimation^34^, individualized treatment learning rule^35^, conditional average treatment effect estimation^36^, as well as uplift modeling^37^. In this work, an application of the uplift modelling based on exact matching is proposed for the causal evaluation of the public policy intervention proposed by INAIL. Such an evaluation aims to estimate the causal impact of the policy with respect to the survival probability of Treated firms. In other words, we aim to measure the ITE obtained by each Treated firm for which a direct public grant was issued for the acquisition of new industrial machineries. Our analysis is based on a binary classification paradigm for which both treatment and outcome values are dichotomous. More formally, let’s consider a dataset D = {(x, y, s)} where x ∈ X represents the observed set of features describing a company characteristic, y ∈ {0, 1} the binary outcome associated to a specific firm (i.e., failure) and s ∈ {0, 1} the corresponding treatment outcome. By considering the Neyman–Rubin potential outcomes^38^ approach, we can define the ITE as follows:

1 $$\tau \left(x\right)={\mathbb{E}}\left[{Y}^{\left(1\right)}-{Y}^{\left(0\right)}|x\right]$$

where Y (1) and Y (0) are the two potential outcomes corresponding to a Treated (s = 1) or non-Treated (s = 0) firm, respectively. The main difficulty in estimating ITE lies in the fact that τ (x) is not directly observable since we can only observe one of the potential outcomes for each company. In fact, a specific firm can be either Treated or be part of the control group but not both. Consequently, this implies that one of the two quantities in Eq. (1) cannot be directly observed, and it represents a counterfactual event. This concept is commonly known in the literature as “*The Fundamental Problem of Causal Inference*”^22^.

In this work, we exploit the randomization between treatment and control firms obtained by the specific policy design. In fact, due to the restricted time window that discriminates firms from being Treated or non-Treated, we can reasonably assume that the characteristics of firms winning the tender resulted no different, on average, with respect to those who did apply but did not receive any grant (i.e., control group). The criterion through which participants were selected is exclusively based on timing. This particular policy evaluation scheme is well known in the econometric literature^39^ due to its proximity to a randomized controlled trial (RCT) experiment, which represents the best policy design for public policy evaluation^40^. In this setting, we can effectively assume that the treatment is randomly provided to each firm participating to the tender, independently from their characteristics *x*. Henceforth, τ (x) can be practically estimated as:

2 $$\widehat{\tau }\left(x\right)=P\left(y=1|x,s=1\right)-P\left(y=1|x,s=0\right)$$

The ITE is thus defined as the difference in probability between firms that go bankrupt given that they received the treatment minus the probability of their respective counterfactual probability estimation, i.e., the probability of bankruptcy had the firm not received any treatment. It is important to realize that either one of these two quantities are observed since, by policy design, a specific firm can either receive the public grant or not but not both (i.e., mutual exclusivity assumption). In this circumstance, the unobservable probability is known as counterfactual. In order to estimate this quantity, an exact matching procedure is implemented. In other words, the characteristics of each Treated firm are matched with those from the control units considering the Mahalanobis Distance Matching (MDM) algorithm. This method requires randomly ordering participants and then calculating the distances between the first Treated participant and all controls^41^. The Mahalanobis metric distance is defined as follows:

3 $$d\left(i,j\right)={\left({v}_{i}-{u}_{j}\right)}^{T}\Phi \left({v}_{i}-{u}_{j}\right)$$

where $i \in N(1)$ and $j \in N(0)$ represent unit indices pertaining to the Treated and control group respectively. Analogously, ${v}_{i}$ represents the set of features of a Treated firm $i$ used for matching to any other control unit $j$ with features vector ${u}_{j}$. Also, $\Phi$ represents the sample variance–covariance matrix of the matching variables. Additionally, two distance functions are also considered for comparison, such as Euclidean distance and cosine similarity. The former is defined as the L2 norm distance between two vectors, and it is mathematically defined as:

4 $${\Vert x-y\Vert }_{2}=\sqrt{\sum_{i}{\left({x}_{i}-{y}_{1}\right)}^{2}}$$

where $|| \cdot |{|}_{2}$ defines the L-2 norm. The Cosine similarity is defined as the dot product of two vector quantities divided by the product of their magnitudes.

5 $${\text{cos}}\left(\theta \right)=\frac{x\cdot y}{\Vert x\Vert \Vert y\Vert }$$

where $|| \cdot ||$ defines the magnitude (i.e., length) of a vector. The difference with respect to the Mahalanobis distance is that the Euclidean distance assumes the data to be isotropically Gaussian. This implies that while the former seeks to measure the correlation between variables, assuming anisotropic Gaussian distribution, the latter Treated all features equally. Conversely, the Cosine similarity measures the angular distance between vectors and is suitable for cases were large number of regressors are available. In order to perform matching, the firm ${x}_{j}$ in the control group with the minimum distance d (${x}_{i}$, ${x}_{j}$), with respect to the Treated unit ${x}_{i}$, is selected, and its prediction used as counterfactual. The process is repeated until at each Treated firm at least one control unit is matched. Notwithstanding, to reduce the dimensionality of the problem and address the high collinearity between regressors, a logistic model with an L1 penalization term is used for feature selection. Specifically, the balance sheet information in the selected years between 2011 and 2014 are fitted with respect to the binary treatment variable and only the covariates with corresponding coefficients different from zero are considered. This approach is already used in other causal inference studies leading to consistent results^42–44^. It should be noted that in order to avoid data leakage, the fitting of the model has to be performed only on the training dataset and the selected feature used for matching Treated units based on the hold out test set. To estimate τˆ the difference in predicted probabilities between matched pairs is performed.

## Statistical and machine learning models

### Linear classifiers: lasso and ridge

In Binary Logistic Regression^45^ models, the relationship between a set of independent variables and a binary dependent variable is estimated. It arguably represents the simplest and most used predictive model for binary classification tasks where the predicted outcome label is obtained by a linear combination between parameters $\theta$ and columns vector $X$. The final probability output $y$ is obtained by passing the latent variable $z$ through a logistic function which maps from a continuous value to a [0,1] range. More formally, the general form of *Logistic Regression* model is as follows:

6 $$z={\theta }_{0}+\sum_{k}{\theta }_{k}{x}_{k}+\epsilon$$

7 $$y=\frac{1}{\left(1+{e}^{-z}\right)}$$

where ${\theta }_{0}$ represents the intercept, $\epsilon$ the residual term and $k\in K$ the regressor column of the data matrix $X$. Notwithstanding, due to the large number of regressors (almost 600 when considering financial scores and other characteristics), such formulation does not allow a proper parameter estimation. For this reason, most often a regularization term will be added. In this study we consider two different types of regularization schemes, such as L1 and L2 regularization, known in the literature as *Lasso* and *Ridge* logistic functions, respectively. Briefly, the L1 penalization term is defined as the sum of absolute values of the regression parameters $\sum_{k}\left|{\theta }_{k}\right|$, while the L2 regularization term considers the sum of squares of the regression coefficients $\sum_{k}{\theta }_{k}^{2}$ (see^46^ for details).

### Boosting models: light gradient boosting machine (LightGBM), extreme gradient boosting machine (XGBoost) and categorical gradient boosting machine (CatBoost)

Unlike many ML models, which focus on high quality predictions done by a single model, boosting represents an ensemble technique that seeks to improve the prediction power by training a sequence of weak models. The main rationale of boosting is to train ML models sequentially in such a way that each subsequent model performs especially well where previous ones failed to achieve a high predictive performance^47^. More precisely, boosting is an ensemble learning technique that uses a set of ML algorithms to convert weak learners into strong learners with the aim of increasing the performance of the final meta-model on a specific task. Boosting algorithms differ in how they create and aggregate weak learners during the sequential stacking process. Gradient boosting exploits differentiable loss functions by using gradient descent approximation to minimize the objective function when adding subsequent learners. Moreover, the function that estimates $\widehat{f}$ is parameterized in an additive functional form as follows:

8 $$\widehat{f}\left(x\right)={\widehat{f}}^{K}\left(x\right)=\sum_{k=0}^{K}{\widehat{f}}_{k}\left(x\right)$$

where $K$ represents the number of iterations, ${\widehat{f}}_{0}\left(x\right)$ represents the starting model and $\{{\widehat{f}}_{k}\left(x\right){\}}_{k=1}^{K}$ defines the set of subsequent learners. The final model $\widehat{f}\left(x\right)$, obtained by sequentially training multiple learners on the residuals of the previous one, is the overall ensemble function. The optimization function is thus defined as:

9 $$\left({\rho }_{t},{\theta }_{t}\right)=\underset{\rho ,\theta }{{\text{argmin}}}\sum_{i=1}^{N}{\left[-{g}_{t}\left({x}_{i}\right)+\rho h\left({x}_{i},\theta \right)\right]}^{2}$$

where $h\left(x,\theta \right)$ represents the subsequent base-learner, $\rho$ defines the learning rate and:

10 $${g}_{t}\left(x\right)={E}_{y}={\left[\frac{\partial \Psi \left(y,f\left(x\right)\right)}{\partial f\left(x\right)} | x\right]}_{f\left(x\right)={\widehat{f}}^{t-1}}$$

while the loss function is defined to be the squared-error (L2 loss):

11 $$\Psi {\left(y,f\left(x\right)\right)}_{{\text{L}}2}=\frac{1}{2}{\left(y-f\left(x\right)\right)}^{2}$$

Most often, a regularization term is added to the final loss function in order to avoid overfitting. In this context, the minimization objective becomes:

12 $$\left({\rho }_{t},{\theta }_{t}\right)=\underset{\rho ,\theta }{{\text{argmin}}}\sum_{i=1}^{N}{\left[-{g}_{t}\left({x}_{i}\right)+\rho h\left({x}_{i},\theta \right)\right]}^{2}+\sum_{k}\Omega \left({f}_{k}\right)$$

where $\Omega \left(f\right)$ represents the regularization term used for penalizing the complexity of the model and is defined in Eq. (13):

13 $$\Omega \left(f\right)= \gamma T+\frac{1}{2}\lambda \Vert \omega \Vert$$

where T defines the number of leaves in the tree and $\gamma$ represents the corresponding weights while the second term in the summation represents the L1 regularization. The additional term added to the final loss helps in smoothing the final learned weights in order to select a model which is the simplest between those that perform the best on the test data. In this work, three of the most widely used and well-performing boosting algorithms are tested for predicting firms’ bankruptcy: Light Gradient Boosting Machine (LightGBM)^48^, Extreme Gradient Boosting Machine (XGBoost)^49^ and Categorical Gradient Boosting Machine (CatBoost)^50^. Each of them exhibits a specific behavior for building trees and dealing with overfitting. In particular, LightBoost uses a novel technique of gradient-based one-side sampling to filter out the data instances to find the optimal split value for building week-learner-trees, while XGBoost implements pre-sorted histogram-based algorithms as a splitting strategy. XGBoost subsamples both rows and columns for training each individual base learner and for the splitting strategy. Finally, CatBoost deals with the problem of prediction shift for which, given a prediction model $F$ the distribution of $F\left(x,k\right)|{x}_{k}$ leads to a shift from the distribution of $F\left(x\right)|x$. In this case, ordered boosting implementation, a modification of standard gradient boosting algorithm, which avoids target leakage, is used.

### Machine learning (survival): random forest (survival), support vector machine (survival) and gradient boosting survival

Although ML model have demonstrated their potential in many predicting tasks, they are not able to deal specifically with survival data. For this reason, modification to the loss function applied to the original algorithms were made available to overcome such limitation. Nonetheless, the inner-working of the algorithms remain unaltered and most often only the loss function is updated. For this reason, it is convenient to describe them jointly.

Specifically, Random Forest Survival (RFSurvival)^51^, represents an ensemble tree-based method for the analysis of right-censored survival data. As is well known, constructing ensembles from base learners, such as trees, can substantially improve prediction performance by artificially injecting noise during the estimation process of each tree. RFSurvival represents an extension of the Random Forest (RF) application proposed by Breiman^50^ for which right-censored outcomes are handled by directly modifying the cost function of each tree in the forest by considering the survival time and censoring information. Analogously to RF, RFSurvival has three main steps. As first step, it draws B bootstrap samples from the original data. In the second step, for each bootstrap sample, a (survival) tree is grown. At each node of a tree, $p$ candidate variables are randomly selected, where $p$ is a parameter, often defined as a proportion of the original number of variables. The task is to split the node into two child nodes using the best candidate variable and split point, as determined by the log-rank test^52^. The best split is the one that maximizes survival differences between the two child nodes. Growing the obtained tree structure is continued until a stopping criterion is met. In the last step, for the survival version of the algorithm, the Cumulative Hazard Function (CHF) associated with each terminal node in a tree is calculated by the Nelson-Aalen estimator, which is a non-parametric estimator of the CHF^53^.

Survival Support Vector Machines (SVMSurvival)^54^ is an extension of the standard Support Vector Machine (SVM) to right-censored time-to-event data. Its main advantage is that it can account for complex, non-linear relationships between features and survival outcome via the so-called kernel trick^55^. A kernel function implicitly maps the input features into high-dimensional feature spaces where the survival function can be described by a hyperplane. This makes SVM extremely versatile and applicable to a wide range of data. The SVMSurvival implementation is based on the original SVM framework proposed by Vapnik^56^ and aims at finding a function that estimates observed survival times as continuous outcome values ${y}_{i}$ given input covariate features $X$. For censored observations, the time to event after censoring is unknown and thus predictions greater than the censoring time do not have to be penalized. However, all survival predictions lower than the censoring time are penalized as usual. For non-censored data, the exact survival times are known and, as in standard SVM, all survival predictions lower or greater than the observed survival time are penalized.

Gradient Boosting Survival (GBSurvival), represents an additional boosting algorithm with the inner working similar to what described in Section "Boosting models: light gradient boosting machine (LightGBM), extreme gradient boosting machine (XGBoost) and categorical gradient boosting machine (CatBoost)". In this paper we followed the modification of the Gradient Boosting Machine implementation proposed by Hothorn et al. whom proposed a unified and flexible framework for ensemble learning in the presence of censoring data, which calculates the negative log-likelihood loss function based on the hazard function represented by a survival tree ensemble^57^.

### Statistical survival models: Cox proportional hazard model, Weibull accelerated failure time, and Aalen’s additive hazard model

The Cox Proportional Hazard Model (CPH)^58^, represents one of the most widely used linear statistical model employed for time-dependent survival analysis. This parametric model extends the univariate version of the Kaplan–Meier estimator^59^ and simultaneously assesses the effect of several risk factors on survival time. In other words, it allows to examine how specific factors influence the rate of a particular event happening (i.e., firm bankruptcy) at a particular point in time. This rate is commonly referred as the *Hazard Rate* (HR). The hazard function is usually denoted as $h\left(t\right)$ and it can be interpreted as the risk of a particular statistical unit of "*dying*" at a specific time $t$. In other words, the hazard represents the expected number of events per one unit of time. Its functional form is defined as follows:

14 $$h\left(t|x\right)={h}_{0}\left(t\right)exp\left(\sum_{i}{b}_{i}{x}_{i}\right)$$

where $t$ represents the survival time, ${b}_{i}$ the *i*-th coefficient of the regression model and ${h}_{0}\left(t\right)$ is called the baseline hazard (identical for each statistical unit) and it corresponds to the value of the hazard if all covariates are set equal to zero. One of the most important limitations of the CPH is the fundamental assumption the Proportional Hazard (PH), which means that the relative hazard remains constant over time with different predictor or covariate levels.

The Weibull Accelerated Failure Time (WeibullAFT)^58^, represents a parametric model that provides an alternative to the commonly used PH models. Whereas a PH model assumes that the effect of a covariate is to multiply the hazard by some constant, an AFT model assumes that the effect of a covariate is to accelerate or decelerate the failure of a firm by some constant. The logarithms of the survival time are considered as a response variable and includes an error term which is assumed to follow a specific probability distribution. More formally, let’s define with $S\left(t\right)$ the survival function at time $t$ and with $\lambda$ an accelerated failure rate parameter. In this case we can formaly write:

15 $$S\left(t\right)={S}_{0}\left(\frac{t}{\lambda \left(x\right)}\right)$$

where ${S}_{0}$ represents the baseline survival function and $\lambda \left(x\right)$ defines the accelerated failure rate estimated based on the covariates observed for each firm, which is defined as follows:

16 $$\lambda \left(x\right)=exp\left({b}_{0}+\sum_{i=1}n{b}_{i}{x}_{i}\right)$$

Thus, this model can accelerate or decelerate failure times depending on subjects’ covariates (${x}_{i}$). Also, by assuming a pre-specified parametric distribution of the survival function $S\left(t\right)$ we can explicitly write the hazard function as follows:

17 $$h\left(t|x\right)={\left(\frac{t}{\lambda \left(x\right)}\right)}^{\rho }$$

This represents a family of distribution that can be fitted to the data by standard maximum likelihood estimation. One of the most important advantages of the AFT model is that it does not requires the PH assumption, which is seldom met, as for the CPH. Interestingly, while in a PH model the covariates act multiplicatively on the hazard, in an AFT model the covariates act multiplicatively on time.

Finally, the Aalen’s Additive Hazard model (AAH)^60^, an additional non-parametric model, represents an alternative to the CPH when the PH assumption is not met. Compared to CPH, in which the linear model is multiplicative, the AAH model considers an additive functional form. One major advantage of such an approach is that effects of covariates are allowed to vary freely over time. Specifically, the standard CPH gives no information about how the effects change over time and valuable information may be lost. On the contrary, the AAH model represents an extension of the Nelson-Aalen estimator, which allows to incorporate covariate-based confounders into the model to estimate the hazard function. More formally, the AAH model is defined as follows:

18 $$h\left(t|x\right)={b}_{0}\left(t\right)+\sum_{i=1}n{b}_{i}\left(t\right){x}_{i}$$

whereby ${b}_{i}\left(t\right)$ represents the regressor coefficient at time $t$. Notwithstanding, similarly to the Nelson-Aalen estimator, these coefficients are estimated considering ${\int }_{0}^{t}{b}_{i}\left(s\right)ds$.

## Experimental set-up

### Model training and parameter instantiation

In this work, in order to calculate the probability of corporate failure, a stratified 10-folds Cross-Validation (CV) procedure is implemented. Specifically, the original dataset is first divided in 10 equal folds. At each fold, the original class imbalance rate is maintained to avoid distortions during the evaluation phase. Then, iteratively, 9 folds are used for model training while the remaining fold (hold-out set) is used for predicting the probability of firm bankruptcy. The entire process is repeated until when a probability is assigned to each firm. We used CV aggregation or crogging^61^, to improve the generalization error estimate using our validation methods. Crogging involves aggregating all validation set predictions (rather than the validation metrics) and computing one validation metric for the entire CV procedure. The entire process is repeated 30 times and the obtained predictions averaged at the instance level in order to obtain reliable results. For all ML models, the same folds partitioning is used in order to guarantee fair comparisons. Also, it is worthwhile to mention that due to the small sample size and high imbalance between classes, no hyperparameter tuning has been employed and the default hyperparameter values for each ML model are used instead, following the approach proposed in previous studies^32,62^. Notwithstanding, cost sensitive learning is applied to deal with class imbalance by re-weighting the loss function toward the less represented (i.e., minority) class^63^. Additionally, for all linear models, both classification and survival-based models, a penalization coefficient of 0.9 is included in order to avoid multicollinearity problems that impede the maximum likelihood convergence, as suggested in Simon et al.^64^. For all tree-based models, 100 trees are used in order to guarantee fair comparison and avoid overfitting, leaving all other parameters at their default values as suggested in Lombardo et al.^32^. Finally, for both SVM models, a Radial Basis Function (RBF) kernel is considered due to the better performances compared to a linear-kernel^65^. The analysis performed in this study are based on Python v3.6 programming language while the lifelines v0.27.4 package is used for survival modeling.

### Performance evaluation

#### Classification metrics

To evaluate the performance of each ML model, different metrics are used based on the task in hand. Specifically, for the binary classification task, the primary performance metric considered in this study is the Area Under the Receiver Operating Characteristic Curve (AUC). This is mostly due to its frequent use in the literature to compare the performance of models on imbalanced datasets and to evaluate the bankruptcy models in general. Such AUC score measures the ability of a classifier to distinguish between classes and is used as a summary of the Receiver Operating Characteristic (ROC) curve. The ROC curve is created by plotting the True Positive Rate (TPR) against the False Positive Rate (FPR) at various threshold settings. An additional evaluation metric used in this study is the Negative Log Likelihood (NLL) loss. Such metric represents a cost function often used in ML application and it measures the goodness of our predictions with respect to the true label. In a binary classification task, it can be reduced to the standard binary cross-entropy loss which can be mathematically defined as follows:

19 $$log {\mathbb{P}}=\sum_{i}\left({y}_{i} log {\widehat{y}}_{i}+\left(1-{y}_{i}\right)log\left(1-{\widehat{y}}_{i}\right)\right)$$

where ${y}_{i}$ represents the true label for the $i$-th firm and ${\widehat{y}}_{i}$ its corresponding prediction. It is important to clarify that the value of the NLL loss depends on the unit of measure. For this reason, a min–max standardization procedure is implemented to avoid bias due to scale measurement. Specifically, the following formulas is used for standardization:

20 $${x}_{i}^{*}=\frac{\left({x}_{i}-{x}_{min}\right)}{\left({x}_{min}-{x}_{max}\right)}$$

where ${x}_{i}^{*}$ defines the standardized values and ${x}_{min}$ and ${x}_{max}$ the vector min and max value respectively. Additional standard classification metrics are also considered based on the analysis of true positive (TP), true negative (TN), false positive (FP), and false negative (FN) instances classified during test phase^66^. Specifically, we consider F1, Precision, and Recall score as classification measures. Formally speaking, F1 score is obtained combining precision and recall and is defined as $2*\frac{Precision*Recall}{Precision+Recall}$. Precision defines the fraction of retrieved instances that are correctly classified $\frac{TP}{TP+FP}$, and Recall identifies the portion of positive instances that are correctly identified $\frac{TP}{TP+FN}$. For binary classification tasks, recall is also known as Sensitivity. For completeness we also considered Specificity as an additional classification metric which measures the probability of a negative event to be in fact negative and it is defined as $\frac{TN}{TN+FP}$. Finally, as additional classification measure, the Jaccard index, is also used. It represents a statistic that measures the similarity and diversity of sample sets. It is defined as follows:

21 $$J\left(Y,\widehat{Y}\right)=\frac{\left|Y\cap \widehat{Y}\right|}{\left|Y\cup \widehat{Y}\right|}=\frac{\left|Y\cap \widehat{Y}\right|}{2*\left|Y\right|-\left|Y\cup \widehat{Y}\right|}$$

where $\left|\cdot\right|$ is the cardinality function, $\cap$ represents the intersection of the two sets and $\cup$ its union. By design, $0\le J\left(Y,\widehat{Y}\right)\le 1$ while if their intersection is empty $J\left(Y,\widehat{Y}\right)=0$. In a binary classification case, the above expression can be reduced to $\frac{TP}{TP+FP+FN}$.

It is important to clarify that beside AUC and NLL scores, all other classification measures require a binarization threshold to transform probabilities in binary outcomes. Thus, the probability of bankruptcy of firms with risk scores below the threshold are imposed to be zero, one otherwise. In order to perform such a task, the ROC curve is used to find the value that maximize the TPR and minimize the FPR on training data. The obtained value is used later on to convert the predicted probabilities of unseen test data.

#### Survival prediction metrics

For the survival analysis case, instead of measuring the absolute survival time for each instance, a popular way to assess a model is to estimate the relative risk of an event occurring for different instances. The Harrell’s Concordance index (C-index)^67^ is a common way to evaluate a model in survival analysis^68^. The C-index can be interpreted as the fraction of all pairs of subjects whose predicted survival times are correctly ordered among all subjects that can actually be ordered. In other words, it is the probability of concordance between the predicted and the observed survival time. Two subjects’ survival times can be ordered either if both are observed or if the observed time of one is smaller than the censored survival time of the other^69^. Consider a set of observations and prediction values for two different instances, $\left({t}_{1},{\widehat{t}}_{1}\right)$ and $\left({t}_{2},{\widehat{t}}_{2}\right)$, where $t$ and $\widehat{t}$ represent the actual and predicted survival time, respectively. The C-index measures the probability between these two instances as follows:

22 $$C=Pr\left({\widehat{t}}_{1}>{\widehat{t}}_{2}|{t}_{1}>{t}_{2}\right)$$

Finally, the Hazard Ratio (HR) represents a measure of how often a particular event happens in one group compared to how often it happens in another group over time and is considered as an additional policy enrichment score. This score is obtained from the CPH model along with its associated *p*-values from log-rank tests. Sample size estimation for CPH assumes a two-sided test and is based on Rosner^70^ and it provides the advantage of taking into account firms that are censored due to reasons other than bankruptcy. Such a score can be used to assess the quality of the ranking between beneficiaries of the policy by dynamically selecting arbitrary thresholds for best responders highlighted by the predictive model.

#### Uplift evaluation metrics

In order to evaluate the impact of the policy intervention at the individual level (ITE), one should ideally compare the predicted uplift with the true value in Eq. (1). Nonetheless, such computation is unfeasible since it involves the notion of a counterfactual, resulting in an unobservable quantity. Radcliffe et al.^71^ proposes to evaluate the goodness of the predicted uplift by computing and plotting the incremental expected uplift value, for an incrementally larger subgroup of the ranked population. The resulting graph is known in the literature as “*Gain Chart*”. Roughly speaking, consider a model that tries to predict a binary outcome (*i.e.,* corporate failure) in a given population. Consider also that the model produces a score for each firm in a way that higher values mean that the firm has more chances of surviving. In this scenario, one builds a gains chart by first sorting for decreasing score all firms, and then plotting the number of corporate failures (or its percentage) against the percentile of firms targeted. More formally, let’s define with $\widehat{\tau }$ the predicted uplift the dataset $\mathcal{D}\left(X,s,y\right)$ would be ranked by, where $y$ is a Boolean variable indicating whether the firm bankrupts, and $s$ a Boolean variable indicating whether the instance is Treated (*i.e.,* won the tender). Furthermore, let’s define with $\pi$ the decreasing ordering of the dataset.

23 $$\pi \left(\phi \right)=\left[x{\left(\phi \right)}_{i}\ge x{\left(\phi \right)}_{j}\right]\forall i,j\in \mathcal{D},\forall \phi \in \Phi$$

where $\phi$ represents the percentage of the population targeted and $\Phi \in \left[0,1\right]$ its distribution. Thus, a gain chart can be defined as follows:

24 $$V\left(\pi ,\phi \right)=\left[\Pi \left(\phi \right)| \pi \left(\phi \right)\right] \forall \phi \in \Phi$$

where

25 $$\Pi \left(\phi \right)=\frac{{N}_{\phi }^{F}}{{N}_{\phi }^{T}}=\frac{\sum_{\forall i\in \pi \left(\phi \right)}{y}_{i}}{\sum_{\forall i\in \pi \left(\phi \right)}{y}_{i}+\sum_{\forall i\in \pi \left(\phi \right)}\left(1-{y}_{i}\right)}$$

where ${N}_{\phi }^{F}$ and ${N}_{\phi }^{T}$ define the subsets of targeted firms and total firms, respectively, with the $\phi {N}^{k}\times 100\mathrm{\%},k\in \left[F,T\right]$ highest predicted uplifts $\widehat{\tau }$. To be noticed that such an analysis can be performed sorting the dataset $\mathcal{D}$ with respect to either $\widehat{\tau }$ or the predicted probability $\widehat{p}$ obtained from the predictive model. For this reason, $x\left(\phi \right)$ in Eq. (23) is used as a generic term by which the sorting of the dataset is performed. In this paper, the gain chart is used for investigating the relationship between treatment and model prediction. In other words, the sorted ITE estimations ($\widehat{\tau }$) are plotted against the cumulative fraction of Treated firm at each threshold $\pi \left(\phi \right)$. An evolution of the gain chart would consider the cumulative incremental gain calculated at each percentile threshold $\phi$ in place of the default ratio $\Pi \left(\phi \right)$ of targeted firms. This type of graph is known in the literature as Qini Curve^72^. A good uplift model will be able to rank firms likely to respond when Treated (*i.e.,* receive public subsidies), leading to higher uplift values in the early parts of the plot^73^. Mathematically, the Qini curve can be defined as follows:

26 $$Q\left(\phi \right)=\frac{1}{{N}_{\phi }^{T}}\left(\sum_{i\in {N}_{\phi }^{T}}{y}_{i}{t}_{i}+\sum_{i\in {N}_{\phi }^{T}}{y}_{i}\left(1-{t}_{i}\right)/\sum_{i\in {N}_{\phi }^{T}}\left(1-{t}_{i}\right)\right)$$

where $Q\left(\phi =0\right)=0$ and $Q\left(\phi =1\right)$ is the ATE. However, such an approach offers only a visual understanding of the performance of an uplift model. For a more formal evaluation, a single number summarizing the overall model performance would be preferred. The Qini coefficient and the Area Under the Uplift Curve (AUUC) represent two commonly used scores in the literature^74^. The former is a natural generalization of the Gini coefficient to the case of uplift modelling and is defined as the area between the actual incremental gains curve from the fitted model and the area under the diagonal corresponding to random targeting. The latter is obtained by considering the ratio between two areas, (1) the area above the diagonal random line and the cumulative gains chart; (2) the area between the diagonal random line and the optimum curve. More formally, let’s define with $\Upsilon=\alpha {\Pi }^{*}$ the worst-case scenario where the uplift is randomly assigned to all firms participating to the tender, regardless of their characteristics. The symbol ${\Pi }^{*}=\Pi \left(\phi =1\right)$ defines the total ratio of corporate failures (100% of data) and $\alpha$ the slope of the diagonal random line. Conversely, an optimal uplift curve can be obtained when ranking corporate failures before censored firms such that:

27 $$Z\left(\phi \right)=\left[\Pi \left(\phi \right) | [ y{\left(\phi | Fail=True\right)}_{i}\ge y{\left(\phi | Fail=False\right)}_{j}\right]$$

The Qini score can be defined as follows:

28 $$Qini={\int }_{0}^{1}Q\left(\pi ,\phi \right)d\phi -{\int }_{0}^{1}\Upsilon d\phi$$

while the AUUC is defined as follows:

29 $$AUUC=\frac{{\int }_{0}^{1}V\left(\pi ,\phi \right)d\phi }{{\int }_{0}^{1}Z\left(\phi \right)d\phi }$$

In this study we propose an additional evaluation metric called Proportional normalized Area Under the Gain chart (PAUG). Roughly speaking, instead of the optimum curve, which represents a practically unrealistic scenario to obtain, the area under the trapezoid $\Omega$ is considered instead, which represents a more meaningful normalizer, and it is defined as follows:

30 $$PAUG\left(\phi \right)=\frac{{\int }_{0}^{1}V\left(\pi ,\phi \right)d\phi }{{\int }_{0}^{1}\Omega \left(\phi \right) d\phi }$$

where

31 $$\Omega \left(\phi \right)=\left\{\begin{array}{ll}0 &\quad if \phi =0\\ \frac{{N}^{F}}{{N}^{T}} & \quad otherwise\end{array}\right.$$

Intuitively, this last measure compares the predictions obtained from the model with the situation where an average uplift $Q\left(\phi =1\right)$ is assigned at each percentile threshold $\phi$. A Riemann’s method based on the trapezoid rule^75^ is used to numerically approximated the area under the curve.

## Results and discussion

### Survival prediction

The performance of the first two objectives tackled in this work such as (1) binary classification and (2) survival prediction of firm bankruptcy, are evaluated based on the performance metrics discussed in Section "Performance evaluation". Table 4 reports the three threshold-free performance metrics calculated for all ML algorithms.

**Table 4:** Threshold-free performance metrics for classification of bankruptcy firm prediction.

Models	AUC	NLL	C-index
Lasso	0.741 (0.029)	0.597 (0.029]	0.734 (0.028)
Ridge	0.738 (0.029)	0.605 (0.033)	0.731 (0.028)
RF	0.797 (0.026)	0.264 (0.034)	0.788 (0.025)
SVM	0.739 (0.024)	0.270 (0.021)	0.731 (0.023)
LightBoost	0.785 (0.028)	0.251 (0.021)	0.778 (0.027)
XGBoost	0.783 (0.026)	0.330 (0.031)	0.775, (0.025)
CatBoost	0.789 (0.028)	0.245 (0.014)	0.782 (0.027)
RFSurvival	0.802 (0.021)	0.224 (0.014)	0.794 (0.020)
SVMSurvival	0.694 (0.027)	0.544 (0.073)	0.688 (0.026)
GBSurvival	0.780 (0.025)	0.236 (0.019)	0.772 (0.024)
CPH	0.735 (0.030)	0.296 (0.033)	0.727 (0.029)
WeibullAFT	0.733 (0.026)	0.613 (0.446)	0.725 (0.025)
AAH	0.690 (0.030)	0.479 (0.206)	0.686 (0.029)

Seven classification algorithms and six survival models are compared for the discrimination between defaulted and censored firms. Standard errors are reported in parenthesis.

**Figure 2:**
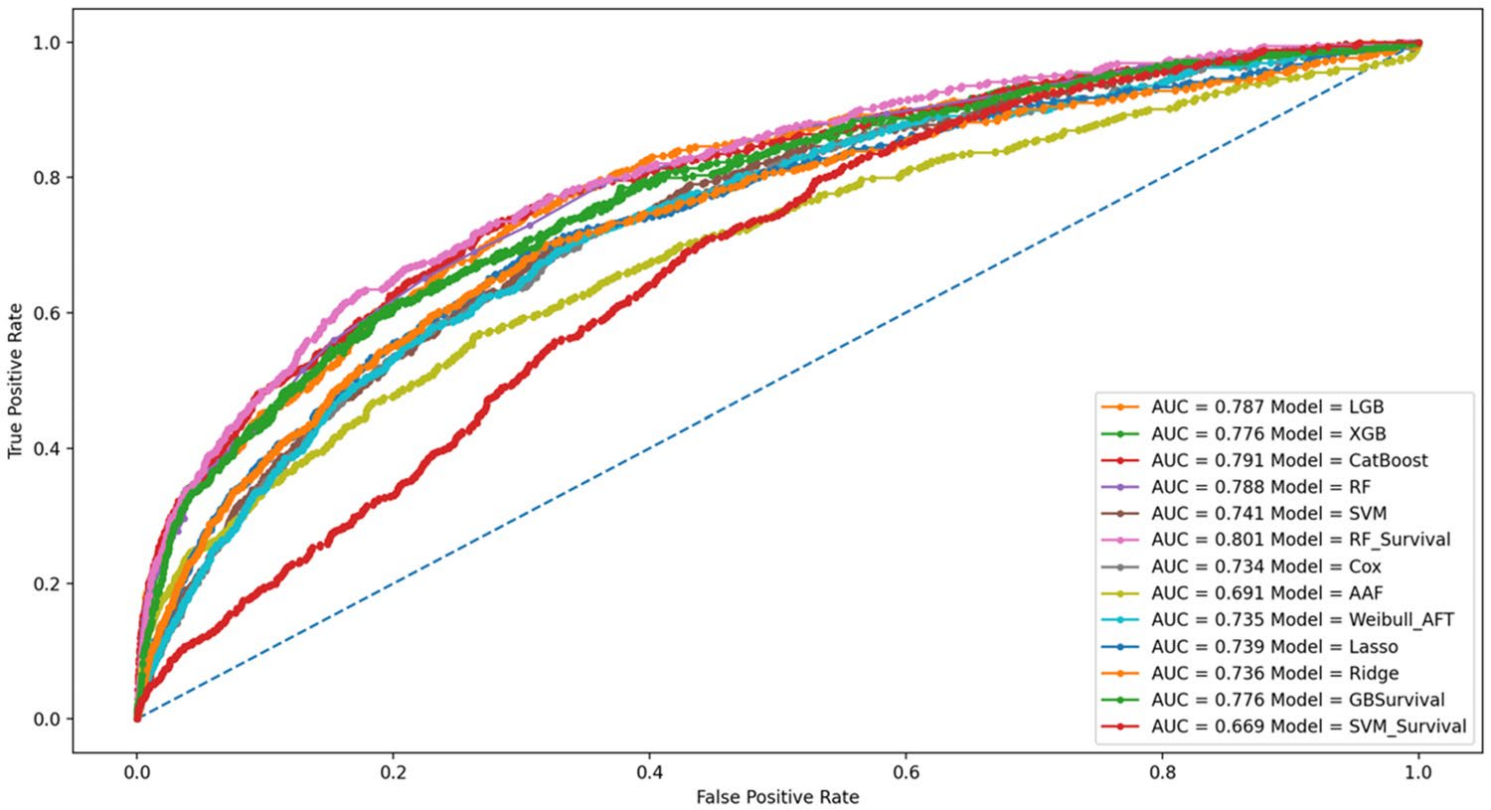
Receiver operating characteristic (ROC) curve.

It is possible to notice that all models resulted with good predictive performances. The highest classification score is obtained with RFSurvival which outperformed all other classification and survival-based algorithms in all threshold-free metrics. Notwithstanding, it is possible to notice that all tree-based models resulted very close to one another and provided the best predictive results, coherently with the literature^32,76^. In terms of C-index and NLL, the same conclusion can be drawn, with a clear advantage for tree-based algorithms. The worst performances are obtained with SVMSurvival and AAF with an AUC score of 0.69 in both cases. The lower performance obtained with the SVMSuvival model can be explained using the RBF kernel which might not be suitable for the current task while the additive component of the AAF model may not reflect the true underline functional form of the data. For a visual comparison of the predictive performance of ML models, the ROC curve is reported in Fig. 2.

In Table 5, the standard binary classification metrics are considered. In terms of $F1$ score, the best performing model results the XGBoost algorithm followed by CatBoost, RF and RFSurvival which all obtained a performance score higher than 0.8. The LightGB model results the only one with a Sensitivity and Specificity score higher than 0.7, highlighting its ability to deal with strong imbalance between classes, even at its default values. The worst predictive performance is obtained by the SVMSurvival model with an $F1$ score of 0.66. This result is mainly due to the low Specificity value obtained by the model, highlighting that when using RBF kernel, the SVMSurvival model is not able to deal properly with class imbalance. In terms of Jaccard score, the XGBoost and CatBoost resulted the only two models with performance scores higher than 0.7.

**Table 5:** Binary classification performance of firms’ bankruptcy.

Models	F1	Precision	Sensitivity	Specificity	Jaccard
Lasso	0.776 (0.015)	0.900 (0.009)	0.713 (0.022)	0.657 (0.050)	0.652 (0.020)
Ridge	0.775 (0.01)	0.902 (0.008)	0.711 (0.015)	0.663 (0.050)	0.651 (0.014)
RF	0.814 (0.015)	0.906 (0.008)	0.770 (0.024]	0.669 (0.057)	0.705 (0.022)
SVM	0.739 (0.025)	0.901 (0.007)	0.657 (0.037)	0.693 (0.056)	0.602 (0.032)
LightBoost	0.785 (0.011)	0.907 (0.008)	0.722 (0.015)	0.709 (0.049)	0.663 (0.014)
XGBoost	0.824 (0.027)	0.902 (0.008)	0.793 (0.044)	0.607 (0.062)	0.722 (0.039)
CatBoost	0.82 (0.024)	0.904 (0.008)	0.783 (0.037)	0.628 (0.055)	0.715 (0.034)
RFSurvival	0.784 (0.017)	0.905 (0.008)	0.723 (0.028)	0.686 (0.058)	0.663 (0.025)
SVMSurvival	0.804 (0.030)	0.906 (0.007)	0.755 (0.049)	0.669 (0.065)	0.692 (0.043)
GBSurvival	0.658 (0.019)	0.901 (0.009)	0.543 (0.026)	0.75 (0.054)	0.501 (0.023)
CPH	0.78 (0.021)	0.898 (0.009)	0.723 (0.034)	0.623 (0.06)	0.658 (0.029)
WeibullAFT	0.775 (0.019)	0.898 (0.009)	0.714 (0.030)	0.630 (0.053)	0.651 (0.027)
AAH	0.723 (0.018)	0.893 (0.010)	0.637 (0.026)	0.650 (0.059)	0.581 (0.023)

Seven classification algorithms and six survival models are compared for the discrimination between defaulted and censored firms. Binarization is performed for each model by considering the threshold of ROC curve that maximize the True Positive Rate (TPR) and minimize False Positive Rate (FPR) in the training dataset. Performance results are obtained applying the optimal threshold on the test set before calculating the performance scores.

The conclusions that can be drawn from this analysis are that for corporate default prediction based on tabular data, tree-based ML models outperform all other statistical and ML algorithms. Overall, our results are in line with the performance obtained in previous studies. For instance, in the study of Lombardo *et al.*^32^ the performance of the XGBoost algorithm ranged from 0.79 to 0.74 in terms of AUC score while for Logistic Regression ranged between 0.86 and 0.69. In Zieba *et al.*^77^ an AUC score between 0.95 and 0.91 is obtained using XGBoost model, while Logistic Regression results with prediction scores between 0.5 and 0.63. Interestingly, in both cases, the SVM model resulted with the worst out-of-sample predictive performance, coherent with our results. In Moscatelli *et al.*^78^, an AUC score between 0.77 and 0.73 is obtained when the XGBoost model is used while the Logistic Regression model results with an AUC score between 0.72 and 0.73. Again, this is coherent with our results suggesting that ML algorithms outperform classical statistical models in out-of-sample prediction. Notwithstanding, to the best of our knowledge, we are not able to find a fair comparison between survival and binary classification models in the context of corporate bankruptcy. Thus, this study may provide a useful benchmark for future applications. In terms of C-index and AUC score we did not find any improvement in using specialized loss function for dealing with censored observations compared to classical discriminative models. However, it is worth emphasizing that for these models, the estimation of the hazard risk is not possible. For this reason, such a measure is substituted with the predicted default probability and the ranking evaluated by considering censored observations. This is a completely valid procedure since the C-index does not take into account the magnitude of the prediction. In fact, its aim is that of measuring the model’s ability to correctly provide a reliable ranking of survival predictions (either hazard-based or probability), adjusting for possible distortions in the ranking due to censored observations.

### Causal impact evaluation

In this section, an evaluation of the public policy intervention designed by INAIL is proposed, which represents the primary aim of this work. In order to estimate the causal impact of the policy on the survival of Treated corporations, both a statistical and ML analysis is developed.

#### Statistical analysis

**Figure 3:**
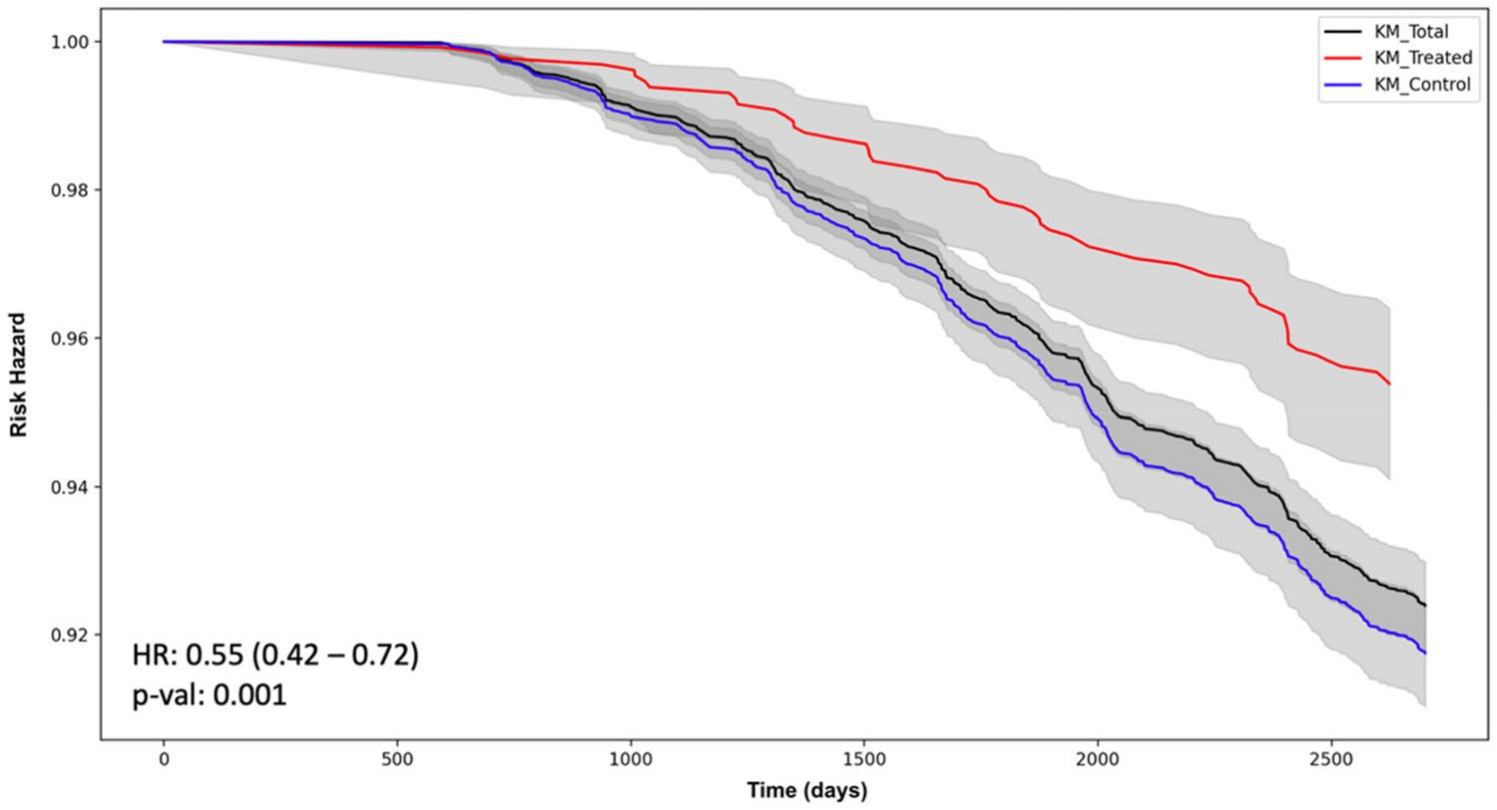
Kaplan–Meier Estimator of the Survival Function between Treated and Control firms with 95% confidence interval (grey shadow). *Note*: Bottom left, Hazard Ration (HR) with confidence intervals in parenthesis.

In order to provide a clear background of the impact of the policy intervention, we start by showing the survival functions of Treated and control firms using the Kaplan–Meier (KM) survival estimator^79^. In Fig. 3, the KM survival functions are depicted in red and blue respectively for the Treated and control units, while in black the survival function for the entire dataset (Treated and non-Treated) is also reported. It is possible to pin-down a clear difference between Treated and control firms when comparing their respective survival functions. Specifically, Treated firms exhibit a less pronounced risk of default compared to controls. The HR results equal to 0.55 with high statistical significance (*p*-value ≤ 0.05).

**Figure 4:**
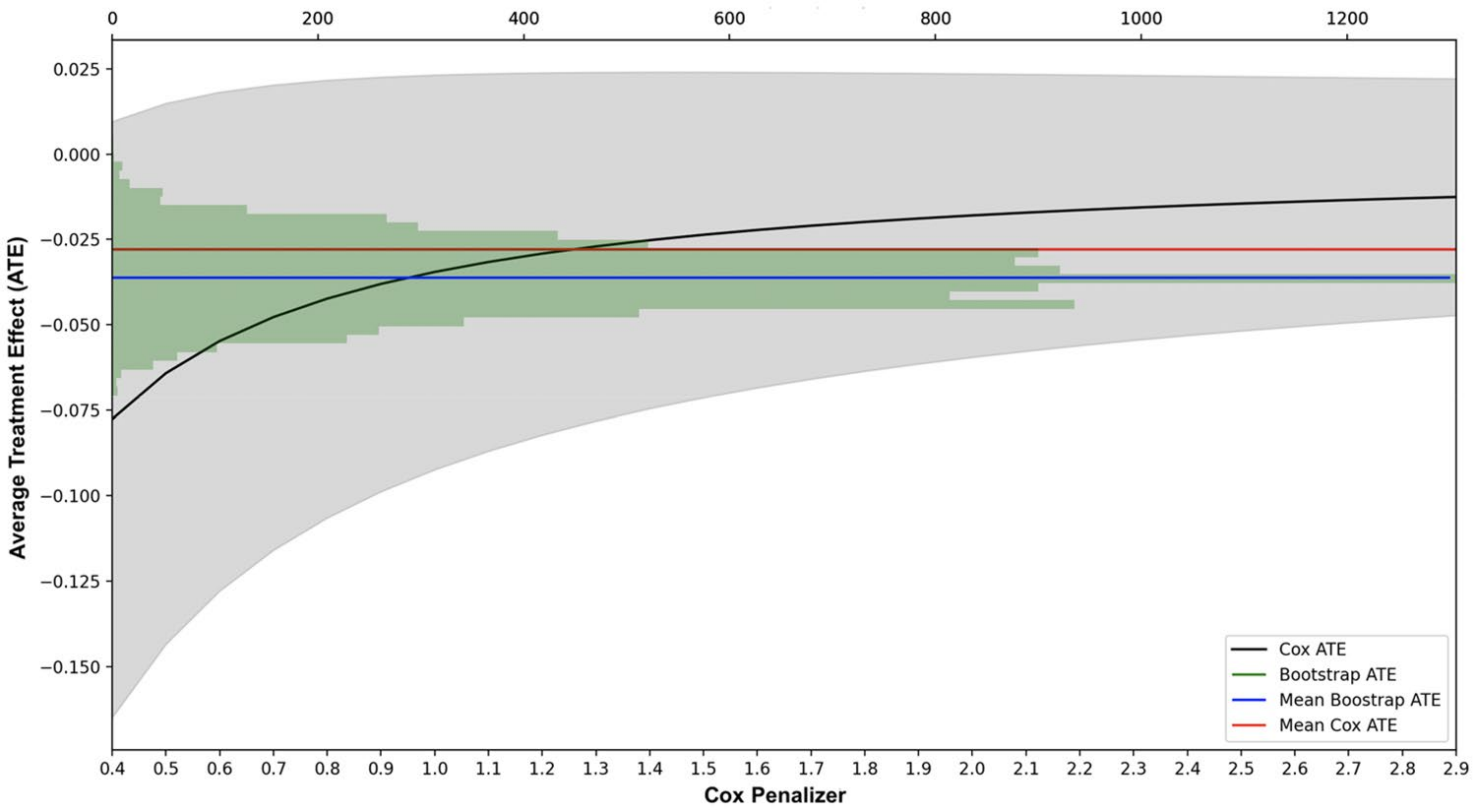
Statistical evaluation of the Average Treatment Effect (ATE). *Note*: A penalized CPH and a Non-Parametric Bootstrap methods are implemented for the estimation of the ATE (vertical axis). For the CPH, different penalization terms in the range between 0.4 and 4 at steps of 0.1 are used (bottom horizontal axis). The black line represents the ATE estimated from the CPH at different penalization terms while in gray the respective 95% confidence intervals. The red line represents the expected ATE over all possible penalization terms. The green histogram depicts distribution of the ATE obtained from the bootstrapping procedure. The frequency for each bin is reported in the upper horizontal axis. The blue line represents the expected ATE of the bootstrapping distribution.

In Fig. 4, two different approaches for the calculation of the ATE are compared. Specifically, the black line represents the CPH (exponential) coefficient obtained using different penalization terms in the range between 0.4 and 3 at steps of 0.1. Penalization terms below 0.4 are avoided due to singularity of the data matrix while penalization values above 3 do not provide additional information. In grey, the upper and lower 95% confidence intervals are also reported. A positive correlation between penalization scores and estimated ATE values can be observed with scores ranging between − 0.075 and − 0.013. The red line identifies the Expected ATE (EATE) obtained by averaging out all ATE scores calculated with different penalization terms, resulting in a value of -0.023. Additionally, a non-parametric ATE estimation is also considered based on a bootstrapping procedure. Specifically, for each group (i.e., Treated and non-Treated), a bootstrap sample is drawn, and the corporate default ratio calculated. The process is repeated ten-thousand times and the difference between the two ratios calculated at each iteration. The ATE distribution obtained through the non-parametric bootstrapped procedure is depicted in green. The blue line represents the mean of the distribution, resulting in a value of -0.036. From this analysis, it is possible to conclude that the policy has effectively contributed to the survival of firms winning the tender. Interestingly, controlling for observable covariates (i.e., CPH) led to a reduction in the EATE score compared to the non-parametric case, although such difference results not statistically significant (*p*-value ≥ 0.05).

#### Uplift modelling: an application

**Figure 5:**
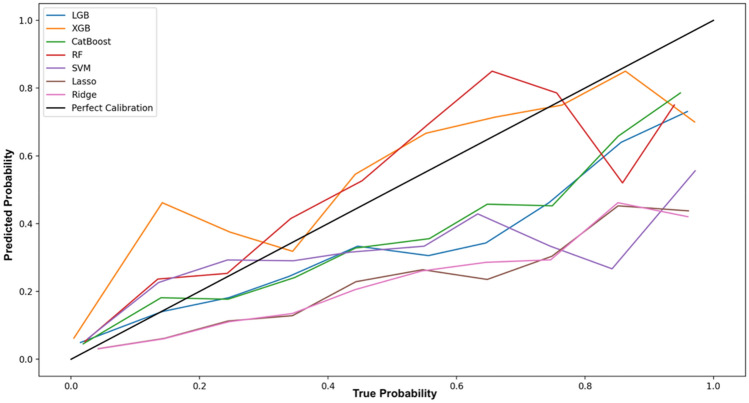
Calibration plot. *Note*: The horizontal axis reports the observed true probabilities while the vertical axis the predicted probabilities considering seven different classification models. The black line represents the optimal case where the predicted and true probabilities are aligned at each decile of the distribution.

Beside statistical approaches, ML-based predictive modelling can provide additional information for a better understanding of the impact of the policy intervention at the individual firm level. As previously stated, in this work, we are interested in calculating the individual uplift score for each firm. First, the Average Treatment Effect (ATE), Average Treatment on the Controls (ATC) and Average Treatment on the Treated (ATT) are calculated by averaging the single uplift scores and compare them to the classical statistical approaches with the aim to check the robustness of the previous results. For a fair comparison, all survival-based models are excluded from the analysis since these types of models do not provide probabilistic interpretation of the results. Additionally, model calibration is required in order to adjust the estimated probabilities to be consistent with what naturally observed. For this purpose, we perform model calibration for each ML model based on the Platt’s calibration method given its good performance with limited data^80^. In Fig. 5, the calibration plot is depicted. It is possible to notice that XGBoost and RF model result well calibrated while a miss-calibration error is still noticeable for all the remaining models. Specifically, for CatBoost and LightGB models, a marginal under-confidence prediction is still present for high probability values while Lasso, Ridge and SVM models remain miss-calibrated for probability values above 0.5. One possible explanation of this findings could be related to the strong unbalance between classes and the small dataset size. However, it is important to highlight that, in the case of sigmoid-based calibration methods, miss-calibration does not affect the quality of the ranking.

In Table 6, the ATE, ATC and ATT estimations are reported. An exact matching procedure is implemented in order to retrieve the ITE for each firm, as explained in Section "Uplift modelling: a theoretical explanation" and thoroughly described in Stuart^81^. Three different distance functions and four neighbor’s groups are considered as additional ablation studies. Seven different classification models are trained to calculate the causal impact of the policy intervention. The results remain stable at different neighbors’ matching groups. Such a result is explained by the low bias in treatment assignment due to the policy design. Nonetheless, ATE resulted different with respect to ATT and ATC probably due to the strong unbalance between groups. However, such discrepancy remains limited and well inside the confidence intervals obtained via bootstrapping. This is particularly important in a context where an almost RCT experiment is performed since matching can reduce model dependence^82^.

**Table 6:** Impact of the policy intervention.

Models	n. Neighbor	Mahalanobis			Cosine			Euclidean		
		ATE	ATT	ATC	ATE	ATT	ATC	ATE	ATT	ATC
Lasso	1	− 0.0486	− 0.0322	− 0.0521	− 0.0112	− 0.0077	− 0.0120	− 0.0112	− 0.0071	− 0.0120
Ridge	1	− 0.0479	− 0.0315	− 0.0515	− 0.0109	− 0.0072	− 0.0117	− 0.0112	− 0.0077	− 0.0120
RF	1	− 0.0154	− 0.0100	− 0.0166	− 0.0232	− 0.0180	− 0.0243	− 0.0248	− 0.0181	− 0.0262
SVM	1	− 0.0119	− 0.0038	− 0.0137	− 0.0116	− 0.0081	− 0.0124	− 0.0114	− 0.0068	− 0.0124
LightBoost	1	− 0.0272	− 0.0185	− 0.0291	− 0.0253	− 0.0171	− 0.0271	− 0.0259	− 0.0131	− 0.0286
XGBoost	1	− 0.0119	− 0.0088	− 0.0125	− 0.0229	− 0.0207	− 0.0234	− 0.0255	− 0.0208	− 0.0265
CatBoost	1	− 0.0275	− 0.0190	− 0.0293	− 0.0236	− 0.0191	− 0.0245	− 0.0256	− 0.0209	− 0.0265
Lasso	3	− 0.0478	− 0.0325	− 0.0511	− 0.0142	− 0.0102	− 0.0151	− 0.0147	− 0.0074	− 0.0163
Ridge	3	− 0.0473	− 0.0320	− 0.0505	− 0.0128	− 0.0093	− 0.0136	− 0.0130	− 0.0101	− 0.0136
RF	3	− 0.0151	− 0.0088	− 0.0165	− 0.0248	− 0.0207	− 0.0257	− 0.0273	− 0.0238	− 0.0280
SVM	3	− 0.0112	− 0.0041	− 0.0127	− 0.0252	− 0.0200	− 0.0263	− 0.0264	− 0.0206	− 0.0277
LightBoost	3	− 0.0258	− 0.0165	− 0.0278	− 0.0105	− 0.0064	− 0.0113	− 0.0105	− 0.0068	− 0.0113
XGBoost	3	− 0.0116	− 0.0063	− 0.0128	− 0.0267	− 0.0216	− 0.0278	− 0.0268	− 0.0164	− 0.0290
CatBoost	3	− 0.0270	− 0.0178	− 0.0289	− 0.0237	− 0.0242	− 0.0236	− 0.0268	− 0.0239	− 0.0274
Lasso	6	− 0.0444	− 0.0332	− 0.0468	− 0.0097	− 0.0047	− 0.0108	− 0.0101	− 0.0045	− 0.0113
Ridge	6	− 0.0438	− 0.0327	− 0.0462	− 0.0419	− 0.0288	− 0.0447	− 0.0465	− 0.0272	− 0.0506
RF	6	− 0.0141	− 0.0080	− 0.0154	− 0.0099	− 0.0059	− 0.0108	− 0.0107	− 0.0057	− 0.0118
SVM	6	− 0.0103	− 0.0041	− 0.0117	− 0.0096	− 0.0050	− 0.0106	− 0.0105	− 0.0050	− 0.0117
LightBoost	6	− 0.0244	− 0.0153	− 0.0264	− 0.0135	− 0.0090	− 0.0145	− 0.0140	− 0.0101	− 0.0148
XGBoost	6	− 0.0110	− 0.0058	− 0.0121	− 0.0131	− 0.0089	− 0.0140	− 0.0130	− 0.0094	− 0.0138
CatBoost	6	− 0.0259	− 0.0169	− 0.0278	− 0.0088	− 0.0045	− 0.0097	− 0.0104	− 0.0032	− 0.0120
Lasso	10	− 0.0450	− 0.0350	− 0.0471	− 0.0432	− 0.0366	− 0.0447	− 0.0449	− 0.0348	− 0.0470
Ridge	10	− 0.0444	− 0.0345	− 0.0465	− 0.0403	− 0.0345	− 0.0415	− 0.0451	− 0.0353	− 0.0472
RF	10	− 0.0138	− 0.0079	− 0.0150	− 0.0418	− 0.0283	− 0.0447	− 0.0458	− 0.0266	− 0.0500
SVM	10	− 0.0103	− 0.0038	− 0.0118	− 0.0422	− 0.0384	− 0.0430	− 0.0466	− 0.0381	− 0.0484
LightBoost	10	− 0.0245	− 0.0160	− 0.0263	− 0.0427	− 0.0389	− 0.0435	− 0.0471	− 0.0387	− 0.0490
XGBoost	10	− 0.0106	− 0.0052	− 0.0118	− 0.0438	− 0.0371	− 0.0452	− 0.0454	− 0.0354	− 0.0476
CatBoost	10	− 0.0257	− 0.0161	− 0.0278	− 0.0410	− 0.0349	− 0.0423	− 0.0457	− 0.0358	− 0.0479

The Average Treatment Effect (ATE), Average Treatment on the Treated (ATT) and Average Treatment Effect on the Control (ATC) is calculated considering seven standard classification algorithms.

**Figure 6:**
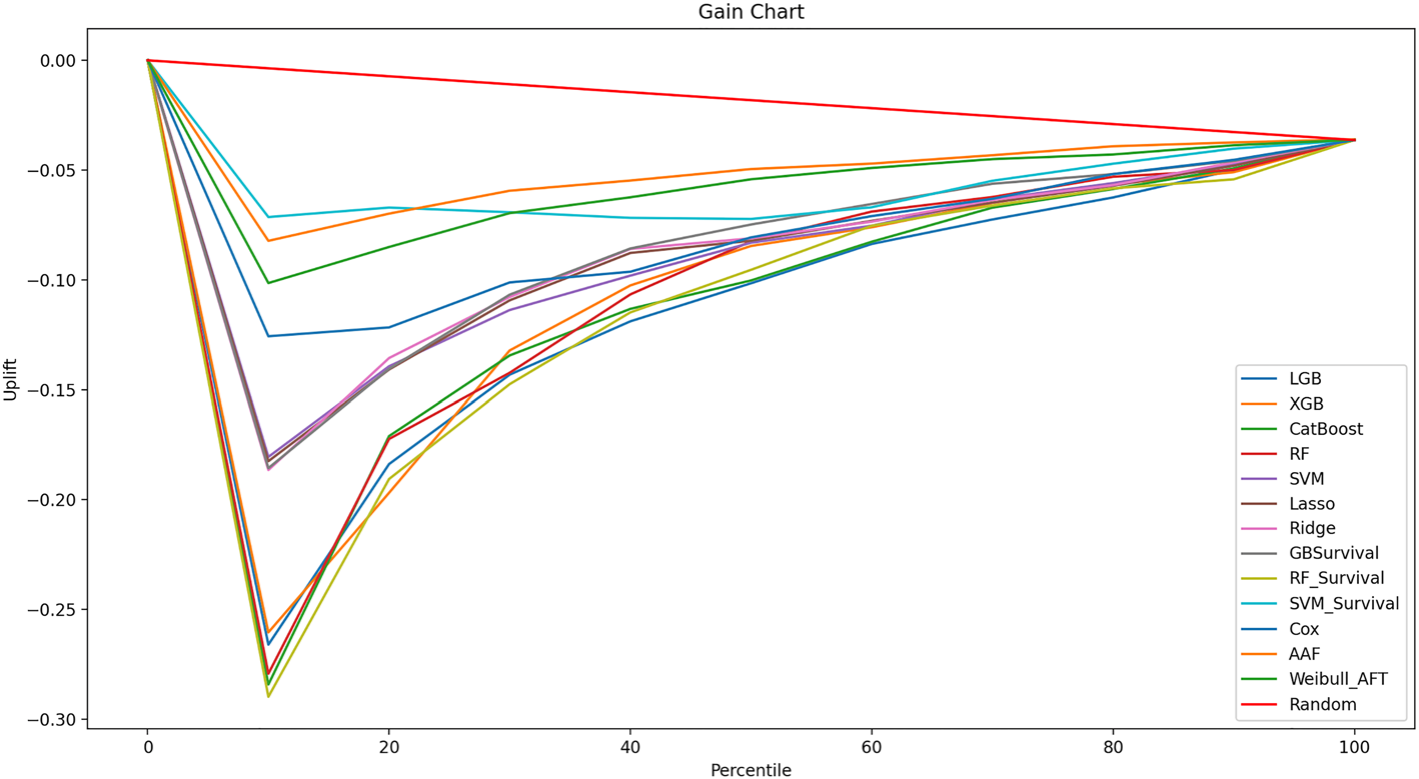
Gain chart for uplift evaluation. *Note*: We present a comparison between seven ML classification models and six survival-based models on the ranking capability of selecting the most responsive firms to the treatment. In the horizontal axis the percentile distribution of the predicted uplift is considered while on the vertical axis the observed cumulative ATE is reported.

This is coherent with our results where the ATE, estimated through the ITE, remained stable in the range be- tween − 0.01 and − 0.05 in almost all cases. Interestingly, Lasso and Ridge models report the highest absolute effect with an ATE value almost always equal to − 0.04. Conversely, the XGBoost and SVM model report consistently the smallest effect in absolute terms with an ATE value of − 0.01. However, when the number of neighbors increases to 10 and the distance function changed to Cosine and Euclidean distance, a higher negative ATE score is observed for Lasso and Ridge models. This result suggests that the Lasso and Ridge models may provide too optimistic results in exchange for stability. Nonetheless, estimating ATE from ITE represents a strong limitation since uplift modelling can be used for selecting the most responsive firms. Such an approach has already been investigated in other field of studies^83^, although its implementation in the context of public policy evaluation remains limited. In Fig. 6, the uplift percentile distribution estimated from each model (horizontal axis) is plotted against the ATE estimated at each percentile threshold (vertical axis). Specifically, for each decile of the ITE distribution (horizontal axis), we compute the ATE (vertical axis). It is clear from the image that by sorting the dataset from the most responsive to the least responsive firms, a monotonic increase of the cumulative uplift is observed. Most of the models are able to accurately rank firms with respect to which the policy intervention is more effective, reducing the default probability up to 30% when the 10% of most responsive firms are selected. This is an interesting result since such a percentage represents a 10-folds increase in magnitude with respect to the overall bootstrapping ATE estimation.

Nonetheless, a wide heterogeneity of results is observed between different models with most of the survival-based models lacking behind compared to classical statistical learning approaches with an exception for RFSurvival. To facilitate the comparison between models, in Table 7 three uplift evaluation metrics are considered to assess the ranking reliability. In terms of AUUC, SVMSurvival resulted the least performing model with a score lower than 0.026. Conversely, LightGB results the most performing model with a score of 0.064, followed by CatBoost and RFSurvival with a value of 0.062. The Qini coefficient and PAUG score provided coherent results with scores for the latter always above one. This is important since a monotonic uplift dynamic can be interpreted as a signal of a good uplift model^84^. With the exception of GBSurvival, all tree-based models resulted with a PAUG score higher then 3 suggesting a three-fold increase of the ranking quality compared to the average case where the ATE score is assigned to each individual firm. Additionally, in order to provide a clearer interpretation of the quality of the uplift ranking, the ATE for the upper and lower 20% of the most and least responsive firms, respectively, is reported. For the most responsive group, a large reduction in default probability is observed with values always below -0.10 for most of the models suggesting a higher effectiveness of the policy intervention for this subgroup, with exceptions observed for SVMSurvival, AAF and WeibullAFT models. Conversely, considering the 20% of firms least responsive to the treatment, a positive ATE is obtained, suggesting that for these firms the policy is not effective in preventing the financial failure.

**Table 7:** Quantitative measures for uplift evaluation.

Models	Lower 20%	Upper 20%	AUUC	Qini	PAUG
Lasso	− 0.141	0.046	0.046	0.282	2.685
Ridge	− 0.136	0.044	0.045	0.28	2.661
RF	− 0.172	0.024	0.058	0.366	3.242
SVM	− 0.139	0.039	0.047	0.297	2.723
LightBoost	− 0.184	0.061	0.064	0.407	3.454
XGBoost	− 0.197	0.040	0.057	0.361	3.266
CatBoost	− 0.171	0.051	0.062	0.39	3.386
RFSurvival	− 0.191	0.044	0.062	0.402	3.48
SVMSurvival	− 0.067	0.006	0.026	0.156	1.767
GBSurvival	− 0.141	0.074	0.058	0.369	2.581
CPH	− 0.122	0.024	0.041	0.253	2.399
WeibullAFT	− 0.085	0.042	0.038	0.233	1.729
AAH	− 0.070	0.004	0.032	0.192	1.516

The Area Under the Uplift Curve (AUUC), Qini coefficient and Proportional normalized Area Under the Gain chart (PAUG) are considered as measures for assessing the quality of uplift models. The ATE calculation for the most responsive (Lower) and least responsive (Upper) 20% of the targeted firms is included.

### Determining the quality of matching

In order to reliably estimate the ITE, and therefore obtain the ATE, the quality of the matching should be assessed in order to guarantee that the positive overlap assumption between the distributions of the Treated and control groups is met. It is important to point out that, due to the large number of regressors available in the dataset, classical statistical approaches result cumbersome to use. For this reason, a LightGBM model is trained to discriminate between Treated and its corresponding matched counterfactuals using cross-validation. The process is repeated 100 times and the average of all runs is calculated obtaining an AUC score of 0.58. This result suggests that poor discriminative information remain present after matching, increasing the confidence in the causal estimation. Additionally, the absolute standardized mean difference approach proposed in Staffa *et al.*^85^ is also considered. It is a numeric summary that can be calculated for every baseline covariate, whether continuous or binary. It compares each baseline factor between the treatment and the control groups after matching is completed and uses a pooled standard deviation calculation. An absolute standardized mean difference < 0.1 was observed and considered as strong indication of a negligible difference between groups, as suggested in the original paper.

### Understanding the best responders

In order to dive deeper and better understand the characteristics of the 20% best responders, in Table 8 descriptive statistics on the financial status of best responder firms before the policy intervention are reported. Financial indicators resulted statistically significant at the usual threshold < 0.05 are considered based on the distributional means comparison between the subgroup of best responder firms and total population (100% of participants) by means of a statistical *t*-test with bootstrapping variance estimation. For reference, the average value of the 20% of worst responders as well as for the entirety of the population (no model selection) are also reported. For the analysis, only the LightGBM model is selected since it stands out as the model with the highest AUUC score. First, it can be noticed that for most of the selected variables, significant differences are detected comparing best responders to the case in which no selection is performed (total population), suggesting that the proposed approach identifies statistically significant characteristics in the subgroup of firm that mostly benefitted from the policy intervention. The percentage of firms in sectors at high risk of injury, such as Construction, Manufacturing, Transportation and storage (based on the Italian categorization of Ateco 2007) is also included. Coherently with our a-priori expectations, the best beneficiaries are those in the most “*at risk*” categories with 76% of firms while a lower percentage (69%) is reported for the worst responders, suggesting that the policy correctly targeted firms in critical working sectors. Statistics on the financial status of firms suggest that lower profitability and financial stability represent crucial features of best responders. In fact, most of the financial scores resulted coherent with our a-priori knowledge when comparing the best and worst responders. First, in terms of level of debts, although the total monetary amount resulted not statistically significant, the best responders are those firms with a higher debt to equity ratio (3.38) compared to the worst responder (1.92). Best responders are also those with the highest cost of money (7.82) and with less profitable investments. For the latter, a negative net income is observed for the best responders with an average loss of − 47 thousand euros, suggesting that these companies are experiencing losses in the period just before the policy intervention took place and the increase in liquidity may have helped them avoid financial failure. In fact, providing liquidity to financial corporations with high likelihood of financial distress can help the survival of the company even though the primary aim of the policy does not consider survival as the primary objective. This can be seen from financial scores such as Return on Equity (ROE of 2.4), Return on Sales (ROS of 2.1) and Return on Assets (ROA of 1.4), which resulted significantly lower compared to worst responders. This is confirmed when also considering the Earnings Before Interest, Taxes, Depreciation, and Amortization (EBITDA), which describes the cash profit generated by the company’s operations. Again, the best responders are those with a lower EBITDA value (140 thousand euros). In summary, these results confirm that the best responders are those in financial distress and for which liquidity might play an important role to avoid corporate financial collapse.

**Table 8:** Descriptive statistics on the 20% best and worst responders.

Features		Best	Worst	Total
Ateco 2007 (%)		0.76	0.69	0.74
Debt over Equity (Ratio)		3.38	1.92	2.12
Long Term Debt (Index)		0.12	0.21	0.17
Cost of Money (Index)		7.82	6.13	6.56
EBITDA (thousand Euros)		140	215	283
ROE		2.41	9.96	8.34
ROS		2.17	3.97	4.26
ROA		1.41	4.35	4.51
Net Income (thousand Euros)		− 47.08	32.18	53.23

Best and Worst responders are defined considering the lower (best) and upper (worst).

20% of firms sorted by the predicted uplift score.

## Conclusion and main policy implications

Despite the existence of a theoretical link between OSH and firm economic performance, there is still scant empirical literature on the effect exerted by OSH investments on firm survival. In this paper, we evaluate the effectiveness of 2013 INAIL’s direct aid programme to support firms’ investments in safer machinery. Using a unique micro-founded database provided by INAIL and AIDA, we implement both statistical and CML approaches to estimate the effect on firms’ default.

The OSH direct grant policy evaluated in this study highlights a positive and significant effect on the survival of Treated firms. A thorough analysis of the survival of Treated and control firms is performed by tackling three important objectives, namely: (1) the classical discriminative task of firms’ failure classification was performed considering seven of the most used predictive algorithms and the results compared with the existing literature; (2) the analysis was extended beyond the classical discriminative task by considering six survival-based models and the results compared with classical discriminative algorithms; (3) the causal impact of the policy intervention was assessed by employing a Causal ML strategy based on uplift modelling and the results compared with a standard statistical approach, usually adopted in econometric studies. First, for the task of firms’ default prediction, both discriminative and survival models highlighted high performance scores with results in line with what observed in the literature. No major improvements were observed when the cost function is adjusted to consider censored observations. This result suggests that if the interest lies in the ranking of firms more likely to default, more than an accurate estimation of the actual default time, classical discriminative algorithms should be preferred. Second, the causal impact analysis of the ISI aid-scheme, which represents the main objective of this study, highlights a positive effect on the survival of Treated firms. It is interesting to notice that in Fig. 4, a reduction (in absolute term) of the EATE estimation from − 0.036 to − 0.023 is observed after controlling for available covariates, suggesting that a minor level of distortion (bias) is still present. This can also be seen in Table 6 where ATE, ATT and ATC vary slightly due to the strong class unbalance in the estimated outcome of failure. Nonetheless, such bias remains limited and well inside the confidence intervals estimated via bootstrapping. These results suggest that, if possible, the Conditional ATE (CATE), obtained by controlling for observable covariates, should be preferred over a non-parametric ATE estimation. Additionally, in Table 6, RF and XGBoost result the only two well calibrated models for which an ATE, estimated by averaging all the ITE scores, result with a value between -0.015 and -0.012 respectively, moving the ATE estimation even more toward zero compared to the non-parametric approach. This result is consistent with what observed in the CPH, reinforcing the idea that controlling for existing covariates may provide a more reliable ATE estimation by reducing model dependence. Conversely, Lasso and Ridge result as the models with the highest miss-calibration error, moving the ATE estimation away from zero and in the opposite direction compered to RF and XGBoost models. Finally, the analysis was extended by examining the quality of the ranking obtained through the estimation of the ITE of each firm. In Fig. 6, a 10-folds increase in the observed uplift for the 10% most responsive group is observed suggesting that a good ranking was obtained from the predicted ITE estimations. In Table 7, a quantitative comparison between ML models is performed and based on the mostly used uplift evaluation scores, such as AUUC and Qini coefficient. For both, the LightGB resulted the most performing model followed by CatBoost and RFSurvival. Additionally, the PAUG score was also proposed since in our opinion comparing with respect to the average case, in which we assign an ATE to all firms, represents a more intuitive description of the uplift quality since it measures how much information can be added by using ML models compared to the standard unconditional ATE estimation (*i.e.,* baseline). Also, to better understand the characteristics of the best responders, a classical descriptive analysis was proposed highlighting that the best beneficiary of the policy are those firms with high chances of financial distress as highlighted by their financial scores. To the best of our knowledge no other studies assessed a public policy intervention considering the emerging Causal ML approach based on uplift modelling. The use of point-wise predictions of the effectiveness of the policy intervention may shad addition light on the characteristic of firms most responsive and those least responsive compared to an econometric approach based on the standard ATE estimation.

These findings are particularly relevant especially if we consider that there is a general lack of awareness among managers of the economic impact of a healthy and safe working environment. In particular, the statistical risks of accidents occurring are not easy to assess, unlike the explicit costs of accident prevention^17^.

The main finding of this paper is that extending the policy mix in OSH by including, in addition to regulation and enforcement (sticks), direct incentives (carrots), especially in the case of SMEs, could enhance OSH levels and firms’ economic performance.

Finally, this work emphasises the need to disseminate the knowledge of the economic value of OSH. Indeed, managers must be made aware of the impact of tangible investments in OSH on company performance since productivity and its improvement through specific interventions are key elements of the economic attractiveness of OSH investments^11^. This is why legal measures and incentives to support companies need to be complemented by an economic justification to reverse the trend of cutbacks in risk management and company closures due to poor and unsustainable working lives^17^.

## Data Availability

The data that support the findings of this study are provided by the Italian National Institute for Insurance against Accidents at Work (INAIL) but restrictions apply to the availability of these data, which were used under license for the current study, and so are not publicly available. Data are however available from the authors upon reasonable request and with permission of third party.
